# Changes of circulating neuregulin 4 and its relationship with 25-hydroxy vitamin D and other diabetic vascular complications in patients with diabetic peripheral neuropathy

**DOI:** 10.1186/s13098-020-00550-2

**Published:** 2020-05-19

**Authors:** Pijun Yan, Zhihong Zhang, Ying Miao, Yong Xu, Jianhua Zhu, Qin Wan

**Affiliations:** 1 Department of Endocrinology, The Affiliated Hospital of Southwest Medical University, Luzhou, Sichuan 646000 China; 2 Department of General Medicine, The Affiliated Hospital of Southwest Medical University, Luzhou, Sichuan 646000 China

**Keywords:** Neuregulin 4, Diabetic peripheral neuropathy, Diabetic vascular, Complications, 25-hydroxy vitamin D

## Abstract

**Background:**

Neuregulin 4 (Nrg4) is a novel neurotrophic adipokine associated with the development of diabetic peripheral neuropathy (DPN), however, the pathological mechanism remains poorly understood. The purpose of our study was to investigate the association of circulating Nrg4 with DPN and 25-hydroxy vitamin D [25(OH)D], a multifunctional secosteroid hormone that regulates other neurotrophic factors and adipokines gene expression, and other diabetic vascular complications.

**Methods:**

Circulating Nrg4 levels were measured with an ELISA kit in 164 newly diagnosed type 2 diabetes mellitus (nT2DM) patients. The relationship between circulating Nrg4 and DPN and other parameters was analyzed.

**Results:**

Circulating Nrg4 levels were significantly lower in nT2DM patients with DPN than those without, and subjects in the highest quartile of circulating Nrg4 had significantly lower vibration perception threshold (VPT), the prevalence of DPN, the proportion of persons with VPT > 25 V, and significantly higher circulating 25(OH)D (all *P *< 0.01). Moreover, circulating Nrg4 was positively and independently associated with 25(OH)D, and was negatively with VPT (*P *< 0.01 or *P *< 0.05), but showed no associations with the prevalence of peripheral arterial disease, diabetic nephropathy, and diabetic retinopathy (all *P *> 0.05). Additionally,the prevalence of DPN and risk of DPN development were progressively decreased with increasing circulating Nrg4 quartiles, independently of potential confounding factors.

**Conclusions:**

These data demonstrate that decreased levels of circulating Nrg4 might lead to the development of DPN through its close interaction with circulating 25(OH)D not with other diabetic vascular complications. Further prospective studies are needed to identify our findings in these populations.

## Background

Diabetic peripheral neuropathy (DPN), one of the most common diabetic microvascular complications, is characterized by numbness, pain, weakness, and loss of sensation in your hands, feet, or legs associated with peripheral nerve dysfunction. DPN develops quite early during the disease, reaching at least 10–15% and possibly affecting around 50% of all diabetes patients as diabetes duration increases [1, 2]. DPN is a major risk factor for foot ulcers and amputations, resulting in depression, lower life quality, limited mobility, social dysfunction, potential morbidity and mortality, and a huge economic burden [2, 3]. However, to date, there has been no specific prevention and treatment for DPN other than strict glycemic control and symptomatic relief. Therefore, there is an urgent need to early detect and appropriately control novel modifiable risk factors associated with the development of DPN.

Neuregulin-4 (Nrg4) is an emerging multifunctional adipokine that is secreted predominantly by brown adipose tissue (BAT). Nrg4 acts via paracrine, autocrine or endocrine mechanisms by releasing the epidermal growth factor (EGF)-like domain after photolytic cleavage [3, 4]. Numerous studies showed that decreased Nrg4 levels were closely associated with obesity, insulin resistance, diabetes mellitus, dyslipidemia, metabolic syndrome, non-alcoholic fatty liver disease, inflammation, oxidative stress, and macrovascular disease such as coronary artery disease, myocardial infarcts, subclinical cardiovascular disease (CVD) in rodents and humans [3, 5–15], while all above factors have been reported to contribute to the development of DNP [1–3, 16, 17], suggesting indirectly that Nrg4 appears to play a crucial role in the development of DNP.

In accordance with the this hypothesis, it has been reported that Nrg4, a member of neuregulins, may have neuroprotective and neurotrophic effects and can promote neurite outgrowth and the development of neuronal progenitor stem cells, and modulate the growth and elaboration of pyramidal neuron dendrites, and spine formation in the striatum [18–21]. Recently, there is preliminary in vivo evidence that circulating Nrg4 levels were significantly decreased in newly diagnosed type 2 diabetes mellitus (nT2DM) patients with DPN [3], however, the pathological mechanism underlying the relationship between decreased plasma Nrg4 levels and increased risk of DPN remains poorly understood.

Vitamin D is a multifunctional secosteroid hormone that has a key role in bone homeostasis, and its status is the most commonly determined by measuring circulating 25-hydroxyvitamin D [25(OH)D]. There are bodies of evidence showing that adequate status of vitamin D can modulate insulin sensitivity, immune and inflammatory responses, improve endothelial dysfunction and cardiometabolic health parameters [22–24], and vitamin D deficiency was associated with diabetes and its micro- and macrovascular complications such as DPN, diabetic nephropathy (DN), diabetic retinopathy (DR), peripheral arterial disease (PAD), cerebral infarction, and cardiovascular disease and risk factors, such as hypertension, dyslipidemia, and obesity [25–28]. Previous evidence has demonstrated that 25(OH)D can up-regulate other neurotrophic factors, such as neurotrophin-3 (NT-3), nerve growth factor (NGF), and glial cell line-derived neurotrophic factor (GDNF) [29]. Moreover, it has been also reported that adipose tissue is the major site of vitamin D storage. Recent studies have demonstrated that vitamin D receptor (VDR) and vitamin D metabolizing enzymes are expressed in adipocytes [22].

Furthermore, it has been shown that vitamin D regulates adipocyte apoptosis as well as adipokines gene expression such as adiponectin and omentin-1 [22, 30]. Of note, Nrg4 as a good adipocytokine has similar features and functions to adiponectin and omentin-1, and also as a neurotrophic factor exerts neurotrophic and neuroprotective effects [3]. We hypothesized that circulating 25(OH)D is inversely associated with plasma Nrg4, and circulating 25(OH)D may partially mediate the association of plasma Nrg4 with the development of DPN.

Therefore, the study was conducted to investigate the relationship between plasma Nrg4 and DPN in a cross-sectional population consisted of 164 Chinese patients with nT2DM. Moreover, we evaluated the potential associations among plasma Nrg4 and circulating 25(OH)D, and other diabetic micro- and macrovascular complications (DR, DN, and PAD).

## Methods

### Study population

164 patients with nT2DM between 44 and 87 years of age, from among inpatients in our department of endocrinology between January 2016 and January 2017, were recruited in the cross-sectional study. T2DM is diagnosed by an oral glucose tolerance test (OGTT) based on American Diabetes Association criteria. In the present study, nT2DM patients were split into two groups: 76 patients with DPN (DPN group) and 88 patients without DPN (no DPN group). All nT2DM patients didn’t receive any lipid-lowering agents and anti-diabetic therapy, including diet control, exercise therapy, hypoglycemic agents and insulin. Excluded were patients with other endocrine disorders such as acromegaly, osteoporosis, thyroid disease, and parathyroid disease, type 1 diabetes, acute complications of diabetes, foot ulcers, limb amputations, cardiovascular and cerebrovascular disease, hypertension, nonalcoholic fatty liver disease, hepatic and renal failure, inflammatory or autoimmune diseases, active infection, cancer, pregnancy or lactation, current treatment with immunosuppressive drugs, systemic corticosteroids, prior history of knee or back surgery, any other etiological cause of peripheral neuropathy (e.g., toxin exposure, hereditary, carpal tunnel syndrome, peripheral polyneuritis, infectious polyneuritis, and vasculitis, cervical spondylosis, and lumbar spondylosis), cigarette smoking, alcohol consumption, and use of any drugs known to interfere with oxidant/antioxidant system and peripheral nerve function.

The study was performed in accordance with the ethical guidelines of the 1975 Declaration of Helsinki. The human research ethics committee of the Affiliated Hospital of Southwest Medical University approved the study, and informed consent was obtained from all nT2DM patients before any study-related procedure.

### Anthropometric and biochemical measurements

Information concerning age, gender, medical history and lifestyle factors (smoking and alcohol consumption) was assessed by a standardized questionnaire. The height, body weight, and blood pressures were measured, as described previously [3]. Based on height and body weight measured, body mass index (BMI) of all patients was calculated.

Blood samples of study subjects were collected in the morning after an overnight fast or 2 h after OGTT. Plasma was obtained by centrifugation at 3500 rpm for 10 min at 4 °C and subsequently stored at −80 °C until analytical processing. Fasting blood glucose (FBG) and postprandial 2 h blood glucose (PBG) were measured by the glucose-oxidase method, and an anion exchange high performance liquid chromatography method was performed to determine levels of glycated hemoglobinA1C (HbA1c) (arkray ELUENT 80A, Japan). Lipid profiles, including total cholesterol (TC), triglyceride (TG), high density lipoprotein cholesterol (HDL-C), low-density lipoprotein cholesterol (LDL-C), and serum creatinine (Scr) were analyzed enzymatically using a 7060 full-automatic biochemical analyzer (Hitachi, Tokyo, Japan). Fasting insulin (Fins) level in plasma was determined using an electrochemiluminescence immunoassay (Roche Elecsys Insulin Test, Roche Diagnostics, Mannheim, Germany). The homeostasis model assessment of insulin resistance (HOMA-IR) was calculated to assess insulin resistance [3]. An electrochemiluminescence immunoassay was used to determine circulating concentration of 25(OH)D. Estimated glomerular filtration rate (eGFR) was calculated using Chronic Kidney Disease Epidemiology Collaboration (CKD-EPI) equa-tions modified by a Japanese coefficient [31]. Urinary microalbumin was measured in a random urine sample using an immunoturbidimetric test, and urinary creati-nine was measured enzymatically. The urine albumin-to-creatinine ratio (UACR) is expressed as milligram of albumin excreted pergram of urinary creatinine. DN was defined as the absence of signs or symptoms of other primary causes of kidney damage, the presence of albuminuria (UACR ≥ 30 mg/g creatinine) or an eGFR < 60 mL/min/1.73 m^2^ [31].

### Measurements of circulating Nrg4

Circulating Nrg4 concentrations were also measured with an ELISA (Aviscera Biosciences, Santa Clara, CA). The detection sensitivity was 0.125-0.25 ng/mL, and the standard linear range was 0.25–16.0 ng/mL. Both intra- and inter-assay CVs were smaller than 10%.

### Foot examination and definition of DR

All nT2DM patients were asked whether they had a history for claudication, and underwent a comprehensive foot examination including assessment of the pedal pulses. Then a diagnostic ankle-brachial index (ABI), measured by a continuous-wave Doppler ultrasound probe (Vista AVS, Summit Co., USA), was performed in nT2DM patients. Patients were diagnosed as having PAD, categorized as one of macrovascular complications, if an ABI value < 0.9 on either limb [32]. All nT2DM patients were asked whether they had symptoms including pain, unpleasant abnormal sensations (sensory disturbance of glove and stocking type distribution, tingling, and burning), and numbness in patients’ feet, legs and upper-limb, and undergone physical examination including ankle and knee reflexes examination. Then, quantitative sensory testing (QST), including assessment of pinprick sensation using a 40 g needle, vibrating perception threshold (VPT) using a neurothesiometer, and light touch perception using a 10-g monofilament, was performed to detect peripheral neuropathy in nT2DM subjects. VPT value of either limb exceeded 25 V (volt) was considered abnormal [3]. Patients were diagnosed as having DPN, as described previously [3].

A Canon CR-2 Digital Retinal Camera was performed to obtain two-field fundus photography of patient’s eyes (Canon Inc., Kanagawa, Japan). The presence of DR was assessed by high-quality fundus photographs and an ophthalmologist who is knowledgeable and experienced in the management and treatment of DR.

### Statistical analysis

All data was analyzed by the SPSS version 20.0 (Chicago, IL, USA). The normal distribution of all data was conducted with the Kolmogorov–Smirnov test, and the Levene homogeneity of variance test was performed to test homogeneity of variance.

Categorical variables are presented as numbers (percentages), otherwise, mean ± standard deviation (SD) was given for continuous variables. Categorical variables between two groups were compared by χ^2^ test. Comparison between two groups was performed with Student’s t test for normally distributed continuous variables or Mann–Whitney U test for nonparametric distributed continuous variables. The subjects were divided into four groups based on circulating Nrg4 quartiles (Q1 < 1.61 ng/mL, 1.61 ng/mL ≤ Q2 < 2.71 ng/mL, 2.71 ≤ Q3 ≤ 3.91 ng/mL, Q4 > 3.91 ng/mL). Statistical difference was assessed using one-way analysis of variance (ANOVA) (continuous variables with normally distribution and homogeneity of variance), or the Kruskal–Wallis test (covariates with nonparametric distribution and/or variance uneven). The association between circulating Nrg4 and other variables was investigated by Pearson or Spearman bivariate correlation analysis; subsequently correlations between circulating Nrg4 levels and other variables were further determined with the partial correlation analysis, adjusting for age, gender, and BMI. Then, the independent variables associated with circulating Nrg4 in nT2DM patients were identified with multiple stepwise linear regression analysis. Model covariates included sex, age, BMI, SBP, DBP, FBG, PBG, HbA1C, FIns, HOMA-IR, TG, TC, HDL-C, LDL-C, UACR, eGFR, Scr, 25(OH)D, ABI, and VPT. All covariates were entered into the model at the same time as there was no evidence of collinearity based on individual variance inflation factor (VIF) < 10, and tolerance statistics > 0.1. Last, multivariable logistic regression analysis was also used to identify the association of circulating levels of Nrg4 with risk of DPN. Odds ratios (OR) and 95% confidence intervals (CI) were estimated. In all statistical tests, a *P* value of < 0.05 was considered to be statistically significant (two sided).

For sample size calculation, we used the following formula:

$$ {\mathbf{N}} = \frac{{\left( {\varvec{q}_{1}^{ - 1} + \varvec{q}_{2}^{ - 1} } \right)\left( {\varvec{t}_{{\varvec{\alpha}/2}} + \varvec{t}_{\varvec{\beta}} } \right)^{2} \varvec{S}^{2} }}{{\varvec{\delta}^{2} }} $$

where N is the total sample size (the sum of the sizes of both comparison groups). In our study, it is assumed that test level α = 0.05, β = 0.1, and the population mean difference between nT2DM patients without DPN group and nT2DM patients with DPN group is 1.5 (δ = 1.5). Also, we assume that the sample size ratio between nT2DM patients without DPN group and nT2DM patients with DPN group is 1:1.5. Accordingly, q1 (nT2DM patients with DPN group) = 1.5/(1 + 1.5) = 0.6, and q2 (nT2DM patients without DPN group) = 1/(1 + 1.5) = 0.4. Previous work has proposed that the SD of Nrg4 for nT2DM patients without DPN group is 1.5 (S = 1.5) [3]. Then, all parameters are included in the above formula, and we get the following result:

$$ N\, = \frac{{\left[ {\left( {0.6} \right)^{ - 1} \, + \,\left( {0.4} \right)^{ - 1} } \right]\,\left( {1.960\, + \,1.282} \right)^{2} \, \times \,2^{2} }}{{1.5^{2} }}\, = \,77.85\,\, = \,78 $$

From the result, we known that the minimum sample size in nT2DM patients without DPN group is 31, and the minimum sample size in nT2DM patients with DPN group is 47. In other words, this study have test efficacy of 0.90 at a two-sided α of 0.05 as long as the sample size in nT2DM patients without DPN group is ≥ 31 and the sample size in nT2DM patients with DPN group is ≥ 47.

## Results

### Circulating Nrg4 levels and other clinical characteristics of studied population

The anthropometric, biochemical and clinical parameters of studied population are shown in Table 1. BMI, age, lipid profiles, blood pressure, plasma glucose, insulin, Scr, eGFR, UACR, ABI, and the prevalence of DN, DR and PAD were similar between the two groups (all *P *> 0.05). When compared with nT2DM patients with no DPN, those with DPN had significantly more men, higher VPT values, and lower levels of circulating Nrg4 and 25(OH)D (*P *< 0.01 or *P *< 0.05).

**Table 1:** Circulating Nrg4 levels and other clinical characteristics between nt2dm patients with and without DPN ($\overline{x}$ ± s)

Variables	No DPN	DPN	*P*
	(n = 88)	(n = 76)	
Men/women	32/56	46/30	0.002
Age (years)	63.90 ± 8.44	63.88 ± 9.93	0.991
BMI (kg/m^2^)	25.02 ± 3.96	26.10 ± 3.47	0.067
SBP (mmHg)	123.45 ± 10.76	125.66 ± 12.78	0.232
DBP (mmHg)	73.68 ± 8.48	74.05 ± 9.84	0.796
FBG (mmol/L)	10.71 ± 3.30	11.38 ± 3.51	0.212
PBG (mmol/L)	18.51 ± 6.34	19.42 ± 5.43	0.329
HbA1c (mmol/mol)	86.84 ± 26.159	85.56 ± 21.95	0.736
HbA1c (%)	10.10 ± 2.39	9.98 ± 2.01	0.736
FIns (μU/ml)	11.23 ± 6.52	10.03 ± 5.60	0.183
HOMA-IR	5.20 ± 3.53	4.97 ± 3.03	0.654
TC (mmol/L)	4.27 ± 1.23	4.62 ± 1.32	0.193
TG (mmol/L)	2.12 ± 1.91	2.17 ± 2.02	0.403
HDL-C (mmol/L)	1.12 ± 0.31	1.13 ± 0.42	0.970
LDL-C (mmol/L)	2.53 ± 1.00	2.80 ± 0.95	0.086
UACR (mg/g)	42.43 ± 49.29	41.71 ± 56.23	0.377
Scr (μmol/L)	68.93 ± 18.43	70.39 ± 19.53	0.622
eGFR (mL/min/1.73 m^2^)	87.06 ± 17.89	89.76 ± 18.23	0.342
VPT (V)	14.19 ± 5.47	24.16 ± 11.55	0.000
ABI	1.03 ± 0.12	1.03 ± 0.15	0.581
25(OH)D (ng/mL)	17.36 ± 5.57	14.51 ± 3.99	0.000
Nrg4 (ng/mL)	3.32 ± 1.62	2.49 ± 1.49	0.001
PAD (n, %)	6 (6.81)	6 (7.89)	0.792
DN (n, %)	37 (42.05)	29 (38.16)	0.613
DR (n, %)	10 (11.36)	10 (13.16)	0.726

Data are mean ± SD

*SD* standard deviation, *BMI* body mass index, *SBP* systolic blood pressure, *DBP* diastolic blood pressure, *FBG* fasting blood glucose, *PBG* postprandial 2 h blood glucose, *HbA1c* glycated hemoglobin A1c, *FIns* fasting plasma insulin, *HOMA-IR* homeostasis model assessment of insulin resistance,* TC* total cholesterol, *TG* triglyceride, *HDL-C* high-density lipoprotein cholesterol, *LDL-C* low-density lipoprotein cholesterol, *UACR* urine albumin-to-creatinine ratio, *Scr* serum creatinine, *25(OH)D* 25-hydroxyvitamin D, *Nrg4* Neuregulin 4, *eGFR* estimated glomerular filtration rate, *VPT* vibration perceptionthreshold,*DN*diabetic nephropathy, *PAD* peripheral arterial disease, *DR* diabetic retinopathy, *DPN* diabetic peripheral neuropathy

### Clinical and biochemical characteristics across quartiles of circulating Nrg4 in all nT2DM patients

Table 2 presents the clinical and biochemical characteristics across quartiles of circulating Nrg4 levels. Circulating levels of Nrg4 and 25(OH)D, VPT values, age, prevalence of DPN, the proportion of individuals with VPT > 25 V and three or more abnormal DPN screening were significantly different across quartiles of plasma Nrg4 (*P *< 0.01 or *P *< 0.05). Compared to subjects in the lowest quartile of circulating Nrg4, those in the highest quartile had significantly lower VPT values, the prevalence of DPN, the proportion of persons with VPT > 25 V and three or more abnormal DPN screening, and significantly higher levels of circulating Nrg4 and 25(OH)D, and older age (*P *< 0.01 or *P *< 0.05). However, no significant differences among the four quartile groups were observed for UACR, eGFR, ABI, and the prevalence of DN, DR and PAD (all *P *> 0.05).

**Table 2:** clinical and biochemical characteristics by quartiles of circulating Nrg4 in all nT2DM patients

Covariates	Quartile 1	Quartile 2	Quartile 3	Quartile 4	P
Sample size	41	41	41	41	
Nrg4 (ng/ml)	1.18 ± 0.31	2.14 ± 0.31**	3.26 ± 0.33**^##^	5.18 ± 1.08**^##$$^	0.000
Men/women	24/17	20/21	15/26	19/22	0.263
Age (years)	65.46 ± 9.70	61.29 ± 7.96*	62.37 ± 8.92	66.44 ± 9.19^$^	0.030
BMI (kg/m^2^)	26.55 ± 3.69	25.79 ± 3.95*	25.02 ± 3.90	24.72 ± 3.39*	0.119
SBP (mmHg)	123.51 ± 11.16	122.56 ± 11.50	124.83 ± 13.19	127.00 ± 10.98	0.351
DBP (mmHg)	72.07 ± 11.29	73.37 ± 9.32	75.73 ± 8.18	74.24 ± 7.05	0.333
FBG (mmol/L)	11.48 ± 3.73	11.79 ± 3.69	10.38 ± 2.83	10.44 ± 3.18	0.140
PBG (mmol/L)	18.82 ± 4.76	19.19 ± 5.92	18.91 ± 7.62	18.82 ± 5.26	0.774
HbA1c (mmol/mol)	84.98 ± 23.16	87.45 ± 20.99	85.63 ± 25.90	86.93 ± 27.21	0.966
HbA1c (%)	9.93 ± 2.12	10.15 ± 1.92	9.99 ± 2.37	10.10 ± 2.49	0.966
FIns (μU/ml)	10.05 ± 6.35	11.71 ± 6.29	10.26 ± 5.32	10.68 ± 6.56	0.624
HOMA-IR	4.76 ± 2.96	6.12 ± 3.85	4.65 ± 2.75	4.84 ± 3.44	0.145
TC (mmol/L)	4.42 ± 1.23	4.37 ± 1.33	4.36 ± 1.18	4.56 ± 1.40	0.893
TG (mmol/L)	2.30 ± 2.69	2.32 ± 1.53	1.55 ± 0.80	2.42 ± 2.21*	0.082
HDL-C (mmol/L)	1.07 ± 0.31	1.10 ± 0.46	1.16 ± 0.32*	1.16 ± 0.36**	0.543
LDL-C (mmol/L)	2.67 ± 0.91	2.51 ± 0.92	2.74 ± 1.03	2.70 ± 1.08	0.731
UACR (mg/g)	39.46 ± 8.70	38.02 ± 6.49	38.07 ± 7.79	52.84 ± 9.54	0.123
Scr (μmol/L)	70.56 ± 21.03	69.83 ± 18.44	65.60 ± 16.44	72.42 ± 19.46	0.417
eGFR (mL/min/1.73 m^2^)	88.76 ± 22.44	89.84 ± 15.55	90.76 ± 16.95	83.89 ± 16.29	0.319
ABI	1.04 ± 0.07	1.05 ± 0.11	1.02 ± 0.15	1.01 ± 0.18	0.880
VPT (V)	27.15 ± 11.37	18.80 ± 10.06**	14.29 ± 5.77**	15.00 ± 6.78**	0.000
25(OH)D (ng/mL)	15.91 ± 5.35	14.11 ± 3.05**	15.76 ± 4.51	18.37 ± 6.15^##^	0.007
DPN, n (%)	28 (68.29)	19 (46.34)	16 (39.02)*	13 (31.71)**	0.006
VPT > 25 V, n(%)	28 (68.29)	10 (24.39) **	3 (7.32**^##^	2 (4.88**^##$$^	0.000
Abnormal pricking sensation, n (%)	34 (82.93)	22 (53.66)	25 (60.98)	22 (53.66)	0.250
Abnormal nylon monofilament, n (%)	8 (19.51)	12 (29.27)	11 (26.83)	11 (26.83)	0.808
Reflex abnormalities, n (%)	6 (14.63)	5 (12.20)	3 (7.32)	2 (4.88)	0.431
Two abnormal DPN screening, n (%)	20 (48.78)	16 (39.02)	15 (36.59)	12 (29.27)	0.340
Three or more abnormal DPN screening, n (%)	8 (19.51)	3 (7.32)	1 (2.44)**	1 (2.44)**	0.012
DN, n (%)	16 (39.02)	18 (43.90)	13 (31.71)	19 (46.34)	0.549
DR, n (%)	4 (9.76)	6 (14.63)	4 (9.76)	6 (14.63)	0.824
PAD, n (%)	3 (7.32)	1 (2.44)	4 (9.76)	4 (9.76)	0.543

*P < 0.05, **P < 0.01 compared with quartile 1 group; ^#^ < 0.05, ^##^0.01 compared with quartile 2 group; ^$^ < 0.05, ^$$^0.01 compared with quartile 3 group

### Association of circulating Nrg4 with 25(OH)D, diabetic vascular complications, and other parameters in study subjects

Next, we analyzed the relationships of circulating Nrg4 levels with 25(OH)D, diabetic vascular complications, other parameters by using simple correlations. The simple correlation analysis demonstrated that circulating Nrg4 levels were positively associated with HDL-C and 25(OH)D, and negatively with BMI, VPT values, the prevalence of DPN, and the proportion of individuals with VPT > 25 V (*P *< 0.01 or *P *< 0.05; Table 3). Also, after adjustment for sex, age, and BMI, this positive association of circulating Nrg4 with of 25(OH)D (*P *< 0.01), and this negative associations between circulating Nrg4 and VPT values, the proportion of individuals with VPT > 25 V, and the prevalence of DPN remained statistically significant (all *P *< 0.01; Table 3). Then, multiple stepwise regressions analysis was used to determine the main determinants of circulating levels of Nrg4. We showed that circulating levels of 25(OH)D, VPT values, SBP, and LDL-C were dependent related factors with circulating Nrg4 (Table 3). The multiple regression equation was:

**Table 3:** Linear correlation and multiple regression analysis of variables associated with circulating Nrg4 in study subjects

Variable	Simple				Multiple		
	R	P-value	Adjusted r	Adjusted P-value	β	Standardized β	P-value
Sex	0.125	0.110	–	–			
Age	0.075	0.340	–	–			
BMI	− 0.165	0.035	–	–			
SBP	0.115	0.142	0.125	0.114	0.021	0.150	0.034
DBP	0.083	0.290	0.104	0.190			
FBG	− 0.123	0.116	− 0.077	0.329			
PBG	− 0.032	0.680	− 0.017	0.834			
HbA1c	0.035	0.653	0.031	0.697			
FIns	0.010	0.894	0.006	0.937			
HOMA-IR	− 0.061	0.440	− 0.032	0.690			
TC	0.110	0.159	0.105	0.184			
TG	− 0.034	0.661	0.042	0.598			
HDL-C	0.148	0.059	0.109	0.169			
LDL-C	0.117	0.136	0.113	0.155	0.245	0.149	0.035
UACR	0.122	0.120	0.103	0.19			
eGFR	− 0.078	0.322	− 0.076	0.337			
Scr	− 0.005	0.947	0.058	0.469			
25(OH)D	0.194	0.013	0.240	0.002	0.070	0.220	0.002
ABI	− 0.011	0.893	0.000	0.997			
VPT	− 0.412	0.000	− 0.360	0.000	− 0.059	− 0.372	0.000
DPN	− 0.276	0.000	− 0.218	0.005			
VPT > 25 V	− 0.506	0.000	− 0.433	0.000			
Abnormal prickingsensation	− 0.078	0.322	0.130	0.100			
Abnormal nylon monofilament	0.017	0.832	− 0.079	0.319			
Reflex abnormalities	− 0.116	0.139	− 0.067	0.401			
DN	0.000	0.999	0.045	0.571			
DR	0.003	0.972	0.014	0.864			
PAD	0.055	0.485	0.010	0.896			

In multiple linear stepwise regression analysis, values included for analysis in nT2DM patients were sex, age, BMI, SBP, DBP, FBG, PBG, HbA1C, FIns, HOMA-IR, TG, TC, HDL-C, LDL-C, UACR, eGFR, Scr, 25(OH)D, ABI, and VPT

$$ \text{Y}_{{\text{Nrg4}}} \, = - 0.274-0.059X_{{\text{VPT}}} \,+ \,0.070X_{{{25}\left( {\text{OH}} \right)\text{D}}} \,+ \,\text{0.021X}_{{\text{SBP}}} \,+ \,{0.245X}_{{\text{LDL-C}}} \,\left( {\text{R}\, = {0.476,}\,\text{R}^{\text{2}} \, = \,{0.207}} \right). $$

### Association between quartiles of circulating Nrg4 and 25(OH)D and risk of DPN in study subjects

To assess whether higher circulating Nrg4 can decrease the risk of development of DPN, multivariate logistic regression analysis was mapped. All subjects were categorized into four quartile groups according to circulating Nrg4. As shown in Table 4, the prevalence of DPN and risk of DPN development were progressively decreased with increasing circulating Nrg4 quartiles (*P* for trend < 0.01 or *P* for trend *P *< 0.05). When compared with those in the lowest quartile, nT2DM patients in the highest circulating Nrg4 quartile had significantly lower risk of DPN development before and after adjustment for gender, age, BMI, blood pressure, lipid profiles, blood glucose, insulin, and the prevalence of DR, DN, and PAD (*P* for trend < 0.05). This association between circulating Nrg4 and risk of DPN development remained significant after further adjustment for 25(OH)D (OR = 0.116; 95% CI 0.027–0.494;*P *< 0.05). Likewise, all subjects were categorized into four quartile groups according to circulating 25(OH)D. The result suggest that the prevalence of DPN and risk of DPN development were progressively decreased with increasing circulating 25(OH)D quartiles (*P* for trend *P *< 0.05). When compared with those in the lowest quartile, nT2DM patients in the highest circulating 25(OH)D quartile had significantly lower risk of DPN development before and after adjustment for gender, age, BMI, blood pressure, lipid profiles, blood glucose, insulin, and the prevalence of DR, DN, and PAD (*P* for trend < 0.05). This association between circulating 25(OH)D and risk of DPN development tended to be statistically significant after further adjustment for Nrg4 (OR = 0.586; 95% CI 0.375–0.918;*P *= 0.098).

**Table 4:** Association between quartiles of circulating Nrg4 and 25(OH)D and risk of DPN in all nT2DM patients

	Quartile 1	Quartile 2	Quartile 3	Quartile 4	P for trend
Circulating Nrg4 level					
Circulating Nrg4 (ng/ml)	1.18 ± 0.31	2.14 ± 0.31	3.26 ± 0.33	5.18 ± 1.08	0.000
Prevalence of DPN	28 (68.29)	19 (46.34)	16 (39.02)	13 (31.71)	0.006
Model 1	1	0.401 (0.163–0.986) 0.047	0.297 (0.120–0.738) 0.009	0.216 (0.085–0.546) 0.001	0.008
Model 2	1	0.443 (0.173–1.134) 0.089	0.384 (0.148–0.996) 0.049	0.250 (0.095–0.659) 0.005	0.040
Model 3	1	0.336 (0.102–1.104) 0.072	0.282 (0.082–0.966) 0.044	0.115 (0.032–0.417) 0.001	0.026
Model 4	1	0.363 (0.109–1.208) 0.099	0.297 (0.085–1.037) 0.057	0.100 (0.026–0.392) 0.001	0.027
Model 5	1	0.304 (0.086–1.071) 0.064	0.213 (0.053–0.865) 0.030	0.116 (0.027–0.494) 0.004	0.047
Circulating 25(OH)D level					
Circulating 25(OH)D (ng/ml)	10.44 ± 2.16	14.25 ± 0.61	16.69 ± 0.68	22.77 ± 4.22	0.000
Prevalence of DPN	21 (51.22)	22 (53.66)	23 (56.10)	10 (24.39)	0.013
Model 1	1	1.103 (0.463–2.624) 0.825	1.103 (0.714–1.704) 0.658	0.675 (0.493–0.923) 0.014	0.026
Model 2	1	1.139 (0.472–2.745) 0.773	1.091 (0.693–1.716) 0.707	0.681 (0.493–0.940) 0.020	0.032
Model 3	1	0.787 (0.287–2.160) 0.642	1.081 (0.627–1.864) 0.779	0.586 (0.396–0.867) 0.007	0.040
Model 4	1	0.662 (0.221–1.982) 0.461	1.060 (0.604–1.860) 0.840	0.564 (0.367–0.868) 0.009	0.028
Model 5	1	0.673 (0.209–2.173) 0.508	1.103 (0.622–1.954) 0.738	0.586 (0.375–0.918) 0.020	0.098

Data are expressed as OR (95% CI) + *P* value, unless stated otherwise

Model 1: crude model

Model 2: Model 1 + sex, age, BMI

Model 3: Model 2 + SBP, DBP, TC, TG, FBG, PBG, HbA1C, FIns, HOMA-IR

Model 4: Model 3 + DR, DN, PAD

Model 5: Model 4 + 25(OH)D or Nrg4

*OR* odds ratio; *CI* confidence interval

### The predictive value of circulating Nrg4 and 25(OH)D in detecting DPN

**Fig. 1:**
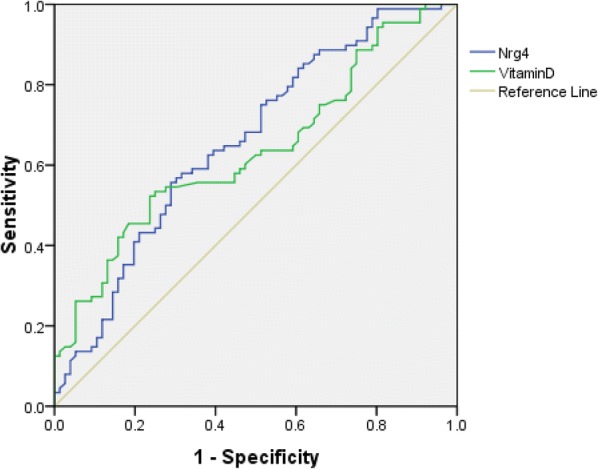
ROC analysis of circulating Nrg4 and 25(OH)D to indicate DPN for nT2DM patients. For circulating Nrg4, AUC = 0.660; 95% CI 0.576–0.744; *P *= 0.000; identified Nrg4 cutoff value = 3.03 ng/ml; Youden index = 0.268; sensitivity: 55.7%; specificity: 71.1%; For circulating 25(OH)D, AUC = 0.635; 95% CI 0.551–0.720; *P *= 0.003; identified 25(OH)D cutoff value = 16.491 ng/ml; Youden index = 0.286; sensitivity: 52.3%; specificity: 76.3%

To explore the predictive value of circulating Nrg4 and 25(OH)D for DPN, we analyzed the ROC curves of circulating Nrg4 and 25(OH)D. The results revealed that the best cutoff value for circulating Nrg4 to predict DPN was 3.03 ng/ml (sensitivity: 55.7%, specificity: 71.1%, and AUC 0.660), and the best cutoff value for circulating 25(OH)D to predict DPN was 16.491 ng/ml (sensitivity: 52.3%, specificity: 76.3%, and AUC 0.635) in patients with nT2DM (Fig. 1).

## Discussion

To the best of our knowledge, this was the first study to illustrate the potential association of circulating Nrg4 with 25(OH)D and other diabetic vascular complications in Chinese patients with nT2DM, and again explore the role of circulating Nrg4 in the development of DPN. We found that circulating Nrg4 significantly decreased in nT2DM patients with DPN, and the risk of DPN was progressively decreased with increasing circulating Nrg4 quartiles even after adjustment for potential confounding factors. More importantly, our study showed for the first time that circulating Nrg4 was positively and independently related to 25(OH)D levels but not related to the prevalence of DN, DR, and PAD.

Nrg4 is a novel and predominantly BAT-secreted adipokine which was upregulated in white fat upon cold exposure [3]. Animal studies have shown that Nrg4 could prevent weight gain, attenuate diet-induced insulin resistance and hepatic steatosis through markedly mitigating hepatic lipogenic signaling, and maintain glycolipid balance [7]. Rosell et al. demonstrated that Nrg4-contained medium from PC12-HER4 cells can promote neurite outgrowth [20]. This was also observed in conditioned medium from transfected Cos7 cells or a chemically synthesized and refolded peptide [21]. Paramo and colleagues performed a study on the striatal Medium spiny neurons (MSN) of Nrg4^+/+^ and Nrg4^−/−^ mice in vivo and in vitro, and showed that Nrg4 was a prominent novel physiological regulator of the formation and maturation of dendritic spines and the growth and elaboration of MSN dendrites [33]. In humans, it has been reported that levels of adipose tissues Nrg4 mRNA and circulating Nrg4 were significantly reduced in T2DM patients compared with healthy individuals [6, 34], and circulating Nrg4 was further markedly decreased in T2DM patients with DPN, and can predict DPN [3, 35]. Consistent with previous studies and our early report [3], we again confirmed that circulating Nrg4 significantly reduced in nT2DM patients with DPN, and was negatively and independently associated with VPT, a useful and reliable marker for early screening DPN and reflecting the clinical severity, and subjects in the highest circulating Nrg4 quartile had significantly lower VPT, the presence of DPN, the proportion of individuals with VPT > 25 V, and three or more abnormal DPN screening as compared to those in the lowest quartile, indicating that decreased circulating Nrg4 may play a crucial role in the pathogenesis of DPN. Moreover, multivariate logistic regression analysis showed that the prevalence of DPN and risk of DPN development were progressively decreased with increasing circulating Nrg4 quartiles, independently of potential confounding factors, further implying a complex mechanistic association between decreased circulating Nrg4 and the development of DPN, however, the exact mechanism of pathogenesis remains unclear.

Several lines of evidence suggest that vitamin D can repair nociceptor function, elevate the pain threshold, modulate neurotransmission, contribute to synaptic plasticity, and has neurotrophic effects on nerve function and nerve growth factors [28]. Previous studies have demonstrated that vitamin D deficiency may play an important role in the development of DPN [26, 28, 36]. Our study provides further evidence that supports the potential role of vitamin D deficiency in the development of DPN, since we found that diabetic patients with DPN had significantly lower circulating 25(OH)D compared with those without, and the prevalence of DPN and risk of DPN development were progressively decreased with increasing circulating 25(OH)D quartiles. More importantly, circulating 25(OH)D was negatively and independently associated with DPN development, and can predict DPN. Interestingly, our study, for the first time, showed that circulating 25(OH)D correlated positively and independently with Nrg4, and circulating 25(OH)D increased along with increasing Nrg4 quartiles, suggesting that there seems to be a close association between circulating Nrg4 and 25(OH)D levels. Experimental studies suggest that vitamin D can up-regulate the synthesis of the neurotrophins such as NGF, GDNF, and regulate neuronal Ca^2+^ homeostasis and neuronal differentiation and maturation [28, 29, 37]. Conversely, low vitamin D levels have been linked to reduced levels of neurotrophins in animal models, with treatment via a vitamin D derivative shown to induce nerve growth factor [38]. Nrg4, member of the EGF family of extracellular ligands, exerts neurotrophic and neuroprotective effects as a neurotrophic factor [3]. Therefore, it is possible that circulating Nrg4 appears to be regulated by 25(OH)D, and lower 25(OH)D may lead to reduced circulating Nrg4. Recent studies have demonstrated that VDR is expressed in adipocytes, and vitamin D regulates adipokines gene expression such as adiponectin and omentin-1 [22, 30]. Gupta et al. showed that a diet rich in VD increased adiponectin synthesis in swine epicardial adipose tissue [39]. Further, a direct effect of 1ɑ, 25-(OH)2 vitamin D3 on adipocytes was demonstrated, with adiponectin and its multimeric forms upregulated by 1ɑ, 25-(OH)2 vitamin D3 treatment at low pharmacological concentrations [24]. Moreover, evidence suggests that, in humans, adiponectin synthesis and secretion is positively controlled by serum 25(OH)D, and circulating 25(OH)D was positively correlated with serum adiponectin [22, 24, 27, 40]. Regarding the effect of vitamin D on omentin-1, Dikker et al. reported that women with normal vitamin D levels had significantly higher serum omentin-1 levels, and circulating vitamin D was positively correlated with serum omentin-1 [30]. Of note, Nrg4 as a good adipocytokine has similar features and functions to adiponectin and omentin, and Nrg4 can promote mRNA expression of several beneficial adipokines, such as adiponectin, adipsin, and vascular endothelial growth factor α [41]. Hence, it is likely that 25(OH)D is effective on the release of Nrg4, and lower circulating 25(OH)D may, at least in part, mediate the association of plasma Nrg4 with the development of DPN. However, further well-designed prospective and larger interventional studies are required to confirm our findings and to better understand the underlying mechanisms.

Numerous studies have shown that vasculopathy may play an important role in the pathogenesis of DPN [17]. As is well known, diabetic vascular complications may have a unifying pathogenic mechanism associated with increased oxidative stress. Slattery et al. and our laboratory demonstrated that levels of Nrg4 gene expression were negatively regulated by oxidative stress [12], and circulating Nrg4 were negatively correlated with markers of oxidative stress (8-iso-prostaglandin F_2α_ and gamma-glutamyltransferase) [3], raising the possibility that circulating Nrg4 may be protective against diabetic micro- and macrovascular complications because of its antioxidant properties, and altered level of circulating Nrg4 may be associated with the development of DN and DR. Surprisingly, we found no significant differences in the prevalence of DN and DR, and DN-related markers across quartiles of circulating Nrg4 and circulating Nrg4 was also not associated with the prevalence of DN and DR, and DN-related markers, suggesting that circulating Nrg4 levels may not involve in the pathogenesis of diabetic microvascular complications other than DPN, and the three diabetic microvascular complications might have other different pathogenetic mechanisms. Our data coincide with a previous report showing that there was no significant difference in serum Nrg4 level between T2DM patients with and without DR [35], but are not consistent with the studies performed by Kralisch et al. and Zahid Kocak et al. [35, 42]. Kralisch et al. found that circulating Nrg4 was significantly lower in patients with end-stage kidney disease on maintenance renal replacement therapy treatment compared to subjects with an eGFR > 50 ml/min/1.73 m^2^ and independently associated with a preserved renal function and mRNA expression of Nrg4 is reduced in adipose tissue depots of mice with diabetic kidney disease as compared to non-diabetic control mice [42]. Similarly, Zahid Kocak and collaborators demonstrted that serum Nrg4 significantly reduced in T2DM patients with DN, and serum Nrg4 was negatively associated with microalbuminuria [35]. The difference between our findings and results of previous studies can be partially explained by selection of different populations, differing sample size, and statistical power. ABI, a surrogate marker for atherosclerosis and predictor of future cardiovascular events, has been widely used to establish the diagnosis of lower extremity PAD, another diabetic macrovascular complication [43, 44]. Our finding that circulating Nrg4 was not related to ABI values and the prevalence of PAD was inconsistent with the results of previous similar studies [13–15]. Jiang et al. found that circulating Nrg4 was inversely associated with subclinical CVD, as evidence by increased carotid intima-media thickness and atherosclerotic plaque in obese adults [13]. A negative correlation between circulating Nrg4 and the presence and severity of coronary artery disease was also reported in another study [14]. Recently, it has been demonstrated that administration of Nrg4 could mitigate the fraction of myocardial infarcts in a mouse model of experimental myocardial ischemia [15]. The discrepant findings between our study and those of previous studies may be partly explained by differing study designs, sample size, selection of patients, duration of diabetes, and medication use. Thus, more evidence is required from longitudinal and randomized controlled studies to validate our findings.

Our study has several limitations that must be acknowledged. Firstly, our results must be carefully interpreted because the cross-sectional study design with small sample size makes it difficult to make any causal inference. Secondly, the present study population consisted of Chinese Han nT2DM patients at a single center, and thus our findings do not apply to other types of diabetes with different ethnicity. Thirdly, 25(OH)D levels affected by many factors, such as dietary supplements, sun exposure, time outdoors, sun-protective behaviors such as use of sunscreen or sun hat, physical activity, season changes, geographic location, sedentary lifestyle and other lifestyle. However, we did not account for these factors because these information is not currently available in our study. Despite this, our study has several advantages. First of all, the DPN was defined using neurological symptoms, reflexes examination, and QST, which were widely used to evaluate whether different types of nerve fibers had neurological dysfunction in clinical screening studies. Secondly, we tentatively explored the relationship between circulating Nrg4 and other diabetic vascular complications, and for the first time found that circulating Nrg4 levels were not associated with the presence of DN, DR and PAD. Last but most importantly, our study is, to our knowledge, the first to evaluate the association between circulating Nrg4 and 25(OH)D in Chinese nT2DM patients, and found that plasma Nrg4 was positively and independently associated with 25(OH)D.

## Conclusions

The present study showed that circulating Nrg4 level significantly decreased in nT2DM patients with DPN, and was independently and negatively correlated with the risk of DPN development. Moreover, circulating Nrg4 levels were independently associated with 25(OH)D, but showed no associations with other diabetic microvascular complications. These findings suggest that decreased circulating Nrg4 may trigger the development of DPN through its close interaction with 25(OH)D not with other diabetic vascular complications. However, our findings need to be confirmed by more well-designed prospective studies.

## Data Availability

The data is available upon reasonable request to the corresponding author.

## Abbreviations

ANOVA: One-way analysis of variance
ABI: Ankle-brachial index
BMI: Body mass index
BAT: Brown adipose tissue
CI: Confidence intervals
CVD: Cardiovascular disease
CKD-EPI: Chronic kidney disease epidemiology collaboration
DPN: Diabetic peripheral neuropathy
DBP: Diastolic blood pressure
DN: Diabetic nephropathy
DR: Diabetic retinopathy
DKD: Diabetic kidney disease
eGFR: Estimated glomerular filtration rate
EGF: Epidermal growth factor
ESKD: End-stage kidney disease
FBG: Fasting blood glucose
Fins: Fasting insulin
GDNF: Glial cell line-derived neurotrophic factor
HDL-C: High-density lipoprotein cholesterol
HbA1c: Glycated hemoglobin A1c
25(OH)D: 25-hydroxy vitamin D
HOMA-IR: Homeostasis model assessment of insulin resistance
LDL-C: Low-density lipoprotein cholesterol
MSN: Medium spiny neurons
Nrg4: Neuregulin 4
nT2DM: Newly diagnosed type 2 diabetes mellitus
NGF: Nerve growth factor
OGTT: Oral glucose tolerance test
PAD: Peripheral arterial disease
PBG: Postprandial 2 h blood glucose
Q: Quartile
QST: Quantitative sensory testing
RRT: Renal replacement therapy
SD: Standard deviation
SBP: Systolic blood pressure
Scr: Serum creatinine
TC: Total cholesterol
TG: Triglyceride
UACR: Urine albumin-to-creatinine ratio
VPT: Vibration perception threshold
